# Should We Be Worried About *Clostridioides difficile* During the SARS-CoV2 Pandemic?

**DOI:** 10.3389/fmicb.2020.581343

**Published:** 2020-09-29

**Authors:** Eliane de Oliveira Ferreira, Bruno Penna, Edwin A. Yates

**Affiliations:** ^1^Laboratório de Biologia de Anaeróbios, Departamento de Microbiologia Médica, Instituto de Microbiologia Paulo de Góes- IMPG, Universidade Federal Do Rio de Janeiro- UFRJ, Rio de Janeiro, Brazil; ^2^Laboratório de Cocos Gram Positivos, Departamento Do Microbiologia e Parasitologia, Instituto Biomédico, Universidade Federal Fluminense, Niteroi, Brazil; ^3^Department of Biochemistry and Systems Biology, Institute of Systems, Molecular and Integrative Biology, University of Liverpool, Liverpool, United Kingdom

**Keywords:** microbiome, COVID−19, SARS–CoV-2, epidemiology, *Clostridioides difficile*

## Introduction

The outbreak caused by the novel coronavirus SARS-CoV-2 and its associated symptoms, termed COVID-19 disease, originally in Wuhan, China in 2019, has rapidly become a global pandemic (Park, 2020). The advent of this contagious virus continues to challenge healthcare systems and has impacted the global economy severely. By 14th June 2020, it had been reported that at least 216 countries and territories were witnessing cases, with 7,690,708 confirmed cases and 427 630 deaths. Notably, a significantly higher mortality rate has been observed among patients over 70 years old and the immunocompromised (Guo et al., 2020). Although the SARS-CoV-2 seems to be less virulent than other coronavirus family viruses, it still has a high rate of transmissibility and can infect lung cells and enterocytes. In severe cases, the symptoms arise from the immune/inflammatory response, resembling macrophage activation syndrome (MAS), and can include diffuse pulmonary intravascular coagulation, resulting in hypoxia. In the majority of patients, elevated levels of cytokines IL1β, IL-6, and IL-10, and TNFα (Tay et al., 2020) have been reported, and can progress to a so-called “cytokine storm,” multiple-organ failure and death (Chen et al., 2020; Tay et al., 2020). The spread of SARS-CoV-2 resulted in a rapid increase in the admission of patients, requiring artificial respiratory support, often stretching the capacity of healthcare systems and highlighting the urgent need for effective therapeutic strategies. Currently, there are no vaccines or treatments that have been developed specifically for SARS-CoV-2 infections, although vaccines and a number of drugs, both of existing re-purposed and novel medications, are in clinical trials (Chary et al., 2020; Park, 2020).

In a great number of individuals, the infection will cause a sub-clinical illness or, mild upper respiratory tract disease. However, when a patient progresses to severe disease, clinicians have to consider using one of the following options: (i) trial drugs, such as: remidesivir, ribavirin, favipiravir and others that aim to hinder virus replication indirectly; (ii) corticosteroids to suppress lung inflammation; (iii) the use of biologics targeting some of the cytokines reported to be up-regulated in patients; (iv) the convalescent plasma transfusion therapy; (v) heparin therapy for improving hypoxia, to avoid intravascular coagulation, which may also hinder the invasion of SARS-CoV-2; (vi) zinc, which controls the proliferation of neutrophils, NK cells, macrophages and lymphocytes and the adverse effect of ROS; (vii) antibiotics (azithromycin and doxycycline), commonly used to modulate the inflammation response (Mycroft-West et al., 2020; Guo et al., 2020; Rahman and Idid, 2020; Zhai et al., 2020).

To make things worse, several studies have demonstrated, based on laboratory, clinical and epidemiology research, that secondary bacterial infections can be a complication of respiratory viral infections, increasing morbidity and mortality. Bacterial co-infections were also reported with MERS-CoV patients in intensive care units (Morris et al., 2017). Indeed, during the 12 influenza epidemics, the majority of deaths were due to bacterial infections (Morens et al., 2008; Gill et al., 2010; Morris et al., 2017). One of the reasons that bacterial co-infections occur is that the upper respiratory tract has a diverse microbiota and can harbor opportunistic pathogens including *Streptococcus pyogenes, S. pneumoniae, Haemophilus influenzae, Staphylococcus aureus*, and *Pseudomonas aeruginosa*. While this problem is likely to be most prevalent among the aged and immunocompromised patients, it is by no means restricted to these groups. For example, pneumonia caused by the rare pathogen, *Mycoplasma pneumoniae*, has been reported in a 36-years old man with ventilatory support during SARS-CoV-2 infection in China, by Fan et al. (2020).

Bengoechea and Bamford (2020), have suggested three scenarios concerning SARS-CoV-2/bacterial co-infections: (a) secondary SARS-CoV-2 following bacterial infection or colonization; (b) combined viral/bacteria pneumonia; (c) secondary antimicrobial resistant bacterial (AMR) after SARS-CoV-2. The authors also point out that administering high doses of drugs to modulate the immune response, such as glucocorticoids to decrease inflammatory processes, can predispose them to fatal secondary bacterial respiratory infections. Although the first two perspectives are of great concern, here, we would like to emphasize the need to consider carefully any increased antibiotic use in combating COVID-19 (Bengoechea and Bamford, 2020). This is particularly relevant in light of the appearance of new hypervirulent bacteria, such as *Clostridioides difficile*, which is still responsible for outbreaks in several countries and remains a global health problem. Furthermore, since the likelihood of other viral diseases emerging in the future is high, the development of an integrated and broadly applicable strategy to enable an early assessment of the inter-linked considerations of disease treatment and secondary, bacterial disease, would be desirable.

Antimicrobial agents have been used for decades and have altered medicine significantly, making a major contribution to the control of infectious diseases (Wright, 2007). However, following their discovery and their global over- and misuse, they have also contributed to the appearance of novel resistant microbes and so called “super-bugs” in all niches, including the human gastrointestinal tract microbiome, which is a huge reservoir for bacteria, archaea, fungi and viruses. The human gut microbiome plays an important role in health, by fermenting indigestible food components into absorbable metabolites, producing essential vitamins, removing toxic compounds, competing with pathogens and helping to shape the immune system (Heintz-Buschart and Wilmes, 2018). Most of these functions are interconnected and tightly linked with human physiology. Equally though, this human gut consortium can be influenced by several factors that include antibiotics (Rice O'connor, 2016) and, its overall composition and diversity can be altered, or become unbalanced. Changes in gut microbiota structure and function after antibiotic treatment create a metabolic environment that favors *C. difficile* germination and colonization, associated with infectious diarrhea, which is debilitating to patients and extremely costly, with symptoms ranging from diarrhea to fulminant colitis, toxic megacolon, and death (Farooq et al., 2015; Czepiel et al., 2019). *Clostridioides difficile* infection (CDI) remains among the top five urgent infectious threats according to the Centers for Disease Control and Prevention (CDC).

Nearly every antibiotic has been implicated in the development of CDI, including the drugs metronidazole and vancomycin, which are used for its treatment. The risk for development of CDI is 8- to 10-fold higher during, and in the 4 weeks subsequent to, antimicrobial therapy and 3-fold higher for the next 2 months (Hensgens et al., 2012). COVID-19 patients receive an empirical antimicrobial therapy with moxifloxacin, cefoperazone, or azithromycin (Chen et al., 2020), drugs that are strongly associated with CDI. Apart from antibiotic use, there are other factors associated with CDI, including higher age (>65 years), longer hospitalization, the use of proton pump inhibitors, comorbidities, chemotherapy, chronic kidney disease, and feeding tubes (Bagdasarian et al., 2015). Although some of those risk factors for CDI are also related to higher probability rates of mortality in severe SARS-CoV-2 infection, the limited number of CDI cases reported among COVID-19 patients is somewhat surprising. Both infections can present similar digestive manifestations including diarrhea, nausea, vomiting and abdominal pain, meaning clinicians need to be even more vigilant for potential co-infections with *C. difficile*. Another problem that COVID-19 may cause relates to fecal microbiota transplantation (FMT) (Chiu et al., 2020; Khanna and Pardi, 2020), which is used for recurrent cases of CDI, accounting for 40–60% of cases (Martin and Wilcox, 2016), in particular, for those patients whose treatment failed, which represent 5–10% of cases (Vigvari et al., 2015). FMT is based on stools donated by volunteers, which are screened and so are considered healthy donors Although FMT seems to reduce the risk for recurrent CDI, the efficacy and safety of this procedure is still under evaluation, especially because the inconsistency found with clinical trials (Wilcox et al., 2020) and deficiency of standards methods for producing FMT (Nicco et al., 2020). In addition to the possible transmission of opportunistic and AMR bacteria, in light of the SARS-CoV-2, all microbiome replacement therapies from now on will require accurate diagnosis to guarantee their safety or, may need to be suspended until the pandemic is over.

## Discussion

To the best of our knowledge, only two clinical surveillances studies reviews have been published. The first one in May 2020, reporting CDI with COVID-19 at the Medical Center in Detroit, Michigan, USA. Patients were screened from 11th March to 11th April, 2020, and in 9 cases received antibiotic therapy, presented diarrhea, experienced SARS-CoV-2 infection and were co-infected with *C. difficile* (Sandhu et al., 2020). The authors emphasized that when CDI is present as a co-infection with COVID-19 and the diarrhea persists, therapy can be difficult because of the SARS-CoV-2 infection. Another concern is the inappropriate use of antibiotics, particularly among patients with mild COVID-19, which could increase the long-term threat of AMR and new epidemic strains. The second one, is from the Saint Michael's Medical Center in Newark (New Jersey, USA), of a 52-year-old man who tested positive for *C. difficile* at admission and presented diarrhea for 3 days. He did not use any drugs correlated with CDI development and had no previous contact with any individuals presenting diarrhea. Besides that, he tested positive for Sars-Cov-2 and was presenting fever, respiratory symptoms and lymphopenia. After he was mechanically ventilated, he received vancomycin and metronidazole, but unfortunately, he died with pneumoniae and septic shock. Authors emphasize that doctors should consider CDI in patients with COVID-19 presenting diarrhea (Lakkasani et al., 2020).

The dearth of studies regarding secondary infections, such as *Clostridioides difficile*, in COVID-19 patients makes it difficult to measure the effect of the pandemic on antimicrobial stewardship programs and on long term antimicrobial resistance. While increased awareness regarding personal hygiene and extensive use of protective equipment may lead to reductions of healthcare associated infections, the challenge of strictly isolating and managing COVID-19 patients in many healthcare systems, often in proximity to patients colonized with *C. difficile*, and the inevitable higher workload imposed on healthcare staff could lead to additional hospital transmissions. The increased use of antibiotics to treat COVID-19 may, inadvertently, have resulted in an under-reporting of *C. difficile* infection. Actually, Spigaglia (2020) has published an article expressing her opinion about the COVID-19 and the impact in elderly patients, who will probably become more susceptible to CDI. The author also demonstrates her concern about the low number of bacterial infections cases related to patients with Sars-Cov-2. To ensure appropriate treatment and to improve patient outcome, increased vigilance and improved diagnosis are both necessary. Given that future emerging viral diseases are highly likely, we would urge increased awareness of the issue and call for informed debate around how to implement effective measures to meet these challenges.

In conclusion, it seems highly likely that cases of CDI are being under-reported among COVID-19 patients and the increased use of antibiotics may, in part, be responsible. To ensure appropriate treatment and improve patient outcome, increased vigilance and improved diagnosis are both necessary. Given that future emerging viral diseases are highly likely, we would urge increased awareness of the issue and call for informed debate around how to implement effective measures to meet these challenges.

## Author Contributions

All authors listed have made a substantial, direct and intellectual contribution to the work, and approved it for publication.

## Conflict of Interest

The authors declare that the research was conducted in the absence of any commercial or financial relationships that could be construed as a potential conflict of interest.
